# Phase II study of gemcitabine, doxorubicin and paclitaxel (GAT) as first-line chemotherapy for metastatic breast cancer: a translational research experience

**DOI:** 10.1186/1471-2407-6-76

**Published:** 2006-03-21

**Authors:** Alessandro Passardi, Ilaria Massa, Wainer Zoli, Lorenzo Gianni, Carlo Milandri, Federica Zumaglini, Oriana Nanni, Roberta Maltoni, Giovanni Luca Frassineti, Dino Amadori

**Affiliations:** 1 Department of Medical Oncology, Morgagni-Pierantoni Hospital, Forlì, Italy; 2 Istituto Oncologico Romagnolo, Forlì, Italy; 3 Department of Oncology, Infermi Hospital, Rimini, Italy; 4 Istituto Scientifico Romagnolo per lo Studio e la Cura dei Tumori, Meldola, Italy

## Abstract

**Background:**

Patients with metastatic breast cancer are frequently treated with anthracyclines and taxanes, which are among the most active agents in this disease. Gemcitabine is an interesting candidate for a three-drug combination because of its different mechanism of action and non-overlapping toxicity with respect to the other two drugs. We aimed to evaluate the activity and toxicity of the GAT (gemcitabine, doxorubicin and paclitaxel) regimen, derived from experimental preclinical studies, as first-line chemotherapy in patients with stage IIIB-IV breast cancer.

**Methods:**

Patients with locally advanced or metastatic breast cancer and at least one bidimensionally measurable lesion were included in the present study. Adequate bone marrow reserve, normal cardiac, hepatic and renal function, and an ECOG performance status of 0 to 2 were required. Only prior adjuvant non anthracycline-based chemotherapy was permitted. Treatment consisted of doxorubicin 50 mg/m^2 ^on day 1, paclitaxel 160 mg/m^2 ^on day 2 and gemcitabine 800 mg/m^2 ^on day 6, repeated every 21–28 days.

**Results:**

Thirty-three consecutive breast cancer patients were enrolled onto the trial (7 stage IIIB and 26 stage IV). All patients were evaluable for toxicity and 29 were assessable for response. A total of 169 cycles were administered, with a median of 6 cycles per patient (range 1–8 cycles). Complete and partial responses were observed in 6.9% and 48.3% of patients, respectively, for an overall response rate of 55.2%. A response was reported in all metastatic sites, with a median duration of 16.4 months. Median time to progression and overall survival were 10.2 and 36.4 months, respectively. The most important toxicity was hematological, with grade III-IV neutropenia observed in 69% of patients, sometimes requiring the use of granulocyte colony-stimulating factor (27%). Non hematological toxicity was rare and mild. One patient died from sepsis during the first treatment cycle before the administration of gemcitabine.

**Conclusion:**

The strong synergism among the three drugs found in the preclinical setting was confirmed in terms of both clinical activity and hematological toxicity. Our results seem to indicate that the GAT regimen is effective in anthracycline-naïve metastatic breast cancer and provides a feasible chemotherapeutic option in this clinical setting.

## Background

Breast cancer is the most common malignancy and the leading cause of cancer mortality in European women. Despite the advances made in treatment options, which have led to a significant increase in survival and quality of life, metastatic disease is still incurable.

Anthracyclines and taxanes are considered the most active drugs for the treatment of metastatic breast cancer (MBC) [1,2], and combination regimens of these agents compared with standard anthracycline-containing chemotherapy in phase III randomized trials [3-9] have shown a higher activity (range of improvement 5–38%) of the former. A significantly higher progression-free survival has generally been observed, whereas only one trial described an important improvement in overall survival [3]. It has therefore been concluded that anthracycline-naive MBC patients who are candidates for chemotherapy should be treated with an anthracycline-containing regimen (possibly in combination with taxanes).

Gemcitabine has shown a high antitumor activity in the treatment of MBC both as single agent and in association with other cytotoxic drugs [10,11]. We hypothesized that paclitaxel, doxorubicin and gemcitabine could be a potentially good combination because of the non-overlapping toxicity, the different mechanisms of action, and the possible lack of cross-resistance of the drugs. In a preclinical study on *in vitro *breast cancer cell lines, we observed a synergistic interaction with the sequence doxorubicin → paclitaxel → 48-h wash-out → gemcitabine [12,13]. On the basis of these results, we then conducted a phase I dose-finding clinical trial on patients with MBC to identify the recommended doses of the drugs [14].

In the present phase II study we aimed to verify whether the strong synergism found in the preclinical *in vitro *model would be confirmed in clinical practice.

## Methods

### Eligibility criteria

Eligibility criteria were as follows: females with histologically and/or cytologically confirmed stage IIIB-IV breast cancer; age between 18 and 70 years; Eastern Cooperative Oncology Group (ECOG) Performance Status 0–2; life expectancy > 12 weeks; adequate cardiac function (left ventricular ejection fraction ≥ 50% by echocardiogram); adequate hepatic and renal function: creatinine and total bilirubin ≤ 1.5 × upper limit of normal, AST e ALT ≤ 3 × upper limit of normal (≤ 5 in patients with liver metastases); adequate bone marrow reserve: absolute neutrophil count (ANC) ≥ 1.5 × 10^9^/L, platelet count ≥ 100 × 10^9^/L, hemoglobin ≥ 9 g/dl. Patients were required to have at least one lesion that was bidimensionally measurable according to WHO criteria, and the presence of only bone metastases was allowed if they were osteolytic lesions and had not previously been treated with radiotherapy. Patients were enrolled irrespective of hormone receptor or menopausal status and HER2/neu expression.

### Exclusion criteria

Exclusion criteria were: active serious infections or severe concomitant diseases (at the discretion of the investigator); known central nervous system tumors, including metastatic brain disease; pregnancy or breast-feeding; previous or concurrent malignancy other than breast cancer, except for non melanoma skin cancer or curatively treated carcinoma *in situ *of the uterine cervix. Prior chemotherapy was limited to one adjuvant non anthracycline-based treatment, and was not permitted for metastatic disease. Previous hormone therapy in an adjuvant setting or for metastatic disease was allowed, provided the patient had progressive disease at study entry. No other anticancer drugs, and only bisphosphonates and palliative radiotherapy of non target lesions were allowed.

All patients gave written informed consent to receive treatment and the study was examined and approved by the Ethics committee of the Local Health and Social Services of each participating center, in accordance with the ethical standards laid down in the 1964 Declaration of Helsinki.

### Treatment plan

The GAT regimen, as defined in the phase I trial, consisted of doxorubicin (A) 50 mg/m^2 ^on day 1, paclitaxel (T)160 mg/m^2 ^on day 2 after 16–24 hours, and gemcitabine (G) 800 mg/m^2 ^on day 6. Cycles were repeated every 21–28 days. Patients were premedicated with methylprednisolone 125 mg i.m. or orally 12 and 6 hours before paclitaxel treatment. Chlorphenamine 10 mg and cimetidine 300 mg were administered intravenously 30 and 15 minutes before paclitaxel, respectively, and antiemetics and colony-stimulating growth factors were administered at the discretion of the investigating physician.

Treatment was given for a maximum of eight cycles and was discontinued in cases of unacceptable toxicity, disease progression, patient refusal, or when, in the judgement of the investigator, a different treatment would be more appropriate for the patient's overall clinical status. In the event of cardiotoxicity or if deemed clinically appropriate, the 7th and 8th cycles could be administered without doxorubicin.

Dose reductions were made in the presence of hematological toxicity (ANC < 0.5 × 10^9^/L and/or platelet count < 50 × 10^9^/L lasting more than 7 days) or grade 3 non hematological toxicity (other than alopecia and nausea/vomiting). Patients were taken off study if grade IV febrile neutropenia, thrombocytopenia with severe bleeding, or any grade IV non hematological toxicity occurred.

### Statistical plan

The primary endpoint of the study was to assess the overall response rate in patients treated with the GAT regimen. Secondary endpoints were toxicity, duration of response, time to progression (TTP) and overall survival (OS).

The trial was conducted using a Simon optimal one-stage design to test the null hypothesis that the response rate was ≤ 30% versus the alternative that it was at least 50%. Thirty-six assessable patients were to be enrolled. If 16 or more objective responses were observed, the regimen would be considered worthy of additional evaluation in this disease. This design yields at least an 80% probability of a positive result if the true response rate is at least 50%, and at least a 95% probability of a negative result if the true response rate is at most 30%.

Efficacy analysis was performed on both intention-to-treat and assessable patient populations. Objective response rates were calculated with 95% confidence intervals. Toxicity analysis was performed on the safety population, i.e. patients having received at least one dose of study treatment. Kaplan-Meyer estimations were used to evaluate response duration, TTP and OS.

### Evaluation of activity and toxicity

Screening evaluation included full medical history, physical and neurological examinations, tumor measurements (by chest x-ray, abdominal ultrasound, or CT scan and bone scan), cardiac function examination (ECG, echocardiogram), hematological and biochemistry analyses including tumor markers CEA and CA15.3, and other evaluations if clinically indicated.

Treatment activity was assessed every two cycles, according to WHO criteria. A complete response (CR) was defined as the disappearance of all lesions and no appearance of new disease for at least 4 weeks. Partial response (PR) was defined as a reduction by at least 50% in the sum of the products of the two longest diameters of all lesions, maintained for at least 4 weeks with no appearance of new disease. CR + PR was rated as the overall response rate. Stable disease (SD) was defined as a less than 50% reduction or less than 25% increase in the sum of the products of the two perpendicular diameters of all measured lesions, with no appearance of new disease. Progressive disease (PD) was an increase of more than 25% in the size of target lesions, or the appearance of an unequivocal new lesion.

Toxicity was evaluated according to WHO criteria after each treatment cycle. Hematochemical assays testing hematological, liver and renal toxicity were performed on days 1, 6 and 15 of each cycle.

Response duration was defined as the interval between the dates of first documented CR, or study entry in the case of PR, and first documented sign of disease progression. TTP was measured from the dates of study entry until disease progression or death, and survival was measured from the dates of study entry until death from any cause.

## Results

### Patient characteristics

Thirty-three consecutive patients with first-line stage IIIB-IV breast cancer from the Medical Oncology Departments of Forlì, Rimini and Ravenna hospitals (IOR group) were entered onto the trial. Patient characteristics are reported in Table 1.

Median age was 59 years (range 35 to 68 years), and the majority of patients were postmenopausal. Median ECOG PS was 0 (range 0 to 1). Seven patients (21.2%) had locally advanced disease, and 17 (51.5%) had more than one metastatic site. Bone or visceral metastases were present in 20 (62.5%) and 19 (59.4%) patients, respectively. Estrogen receptor (ER) status was positive in 22 (66.7%), negative in 8 (24.2%) and unknown in 3 (9.1%) patients.

Sixteen patients (48.5%) had not had antiblastic therapy of any kind, whereas 15 (46.9%) patients had received prior adjuvant chemotherapy and/or hormone therapy, and 6 with advanced disease had been given hormone therapy.

### Treatment activity

Of the 33 treated patients, 29 (87.8%) were assessable for tumor response. One patient was considered ineligible due to the absence of any measurable lesion, whereas 3 patients were not assessable for response because of treatment discontinuation during the first cycle following severe toxicity (febrile neutropenia, anaphylactic reaction to paclitaxel and grade IV diarrhea).

Response rates for the 29 evaluable patients are shown in Table 2. Two patients (6.9%) had a CR and 14 (48.3%) had a PR, for an overall response rate of 55.2% (95 CI, 37.5% to 62.5%). Ten (34.5%) and 3 (10.3%) patients showed SD and PD, respectively. The median response duration was 16.4 months. A response was reported in all metastatic sites and was very high in breast and lymph node sites (75 and 100%, respectively), especially in the 7 stage IIIB patients (100%). Six of the 7 patients had a PR after 4–8 treatment cycles and 5 of these underwent radical mastectomy. The remaining stage IIIB patient obtained a clinical CR and, having refused radical surgery, was treated with adjuvant radiotherapy and endocrine therapy (letrozole). High response rates were also reported in visceral disease (69.2%), but were much lower for liver metastases (27.3%), and none were reported for bone lesions.

Despite preclinical data indicating a stronger synergism and activity in ER- breast cancer cell lines, no differences in response rates were observed between ER+ and ER- breast cancer patients.

**Figure 1:**
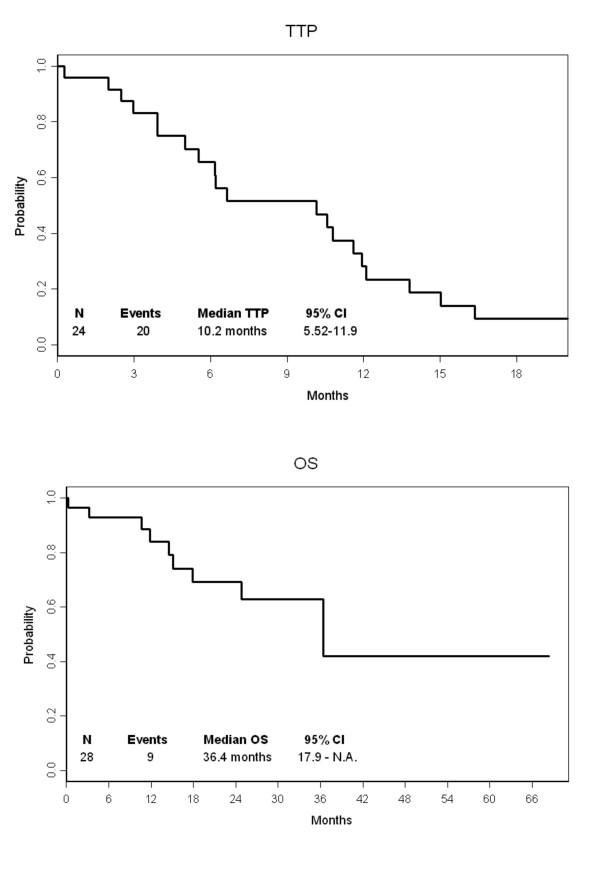
(A) Time to progression (TTP) and (B) overall survival (OS).

TTP and OS evaluation was restricted to the 26 stage IV patients. The median TTP was 10.2 months (Figure 1A). At a median follow-up of 31 months, 10 patients had died and 16 were still alive, with a median OS time of 36.4 months (Figure 1B).

### Treatment safety and toxicity

A total of 169 cycles were administered during the study, with a median of 6 cycles per patient (range 1–8 cycles). Twenty patients (62.5%) received the full number of planned cycles, 15 received 6 cycles, and 5 received 8 cycles. One patient stopped treatment after the 5th cycle to have radiotherapy, and two patients discontinued chemotherapy after the 4th and 5th cycle to undergo radical surgery. Early treatment interruption was required in five patients because of toxicity and in four patients due to disease progression.

The planned timing of chemotherapy was respected for most of the cycles and only 7 (4.2%) cycles were delayed. Logistic problems caused a modest delay (16.6%) on the 6^th ^day of treatment (gemcitabine). A dose reduction in all three drugs was made in 23 cycles (13.6%), whereas doxorubicin-paclitaxel or gemcitabine dose reductions were required in 7 (4.2%) and 9 (5.3%) cycles, respectively. Gemcitabine was not administered in 7 cycles because of toxicity, in 3 due to logistic problems, and in one because of treatment interruption following disease progression (Table 3).

All of the 33 enrolled patients were assessable for toxicity. Toxicities observed per patient and per cycle are reported in Table 4. As expected, the most important toxicity was hematological. Grade 3–4 leucopenia was observed in 42% of patients and 23.6% of cycles, and grade 3–4 neutropenia was recorded in 69% of patients and 48% of cycles. Febrile neutropenia occurred in six patients (18.1%) and nine patients (27.3%) were treated with G-CSF, for a total of 27 cycles (16.6%). One patient died from septic shock during the first treatment cycle, before the first administration of gemcitabine. On the basis of preliminary data showing high hematological toxicity, we tested the prophylactic use of G-CSF in 5 patients (28 cycles overall): no cases of grade III-IV neutropenia were observed and treatment was completed without delays or significant toxicity. Grade 3 thrombocytopenia occurred in 12% of patients and 2.4% of cycles, mild thrombocytosis was observed in 34.3% of cycles, and grade 3 anemia was recorded in 2 patients (6%). There were no cases of grade 4 anemia or thrombocytopenia. Non hematological toxicity was rare and mild. We observed 3 cases of grade 3 toxicity (1 nausea, 1 diarrhea, 1 stomatitis) and one case of grade 4 diarrhea after the first treatment cycle. Neurotoxicity was limited (grade 1–2) and did not require treatment discontinuation. There were no cases of cardiotoxicity. Two patients experienced mild thromboembolism and one patient experienced an anaphylactic reaction to paclitaxel.

## Discussion

Paclitaxel has emerged as an important agent in the treatment of breast cancer. The efficacy and tolerability of this agent, as well as its lack of cross-resistance with anthracyclines, have led to its inclusion in combination treatments [1]. Taxane- and anthracycline-containing regimens have been extensively used, and one prospective phase III clinical study reported a higher response rate and a longer overall survival for paclitaxel-doxorubicin compared to 5-fluorouracil, doxorubicin and cyclophosphamide [3]. These results, however, have been challenged by other authors who did not demonstrate a survival advantage or higher toxicity for taxane-based therapy [4-8]. In view of these inconsistent results, attempts have been made to find schedules that are capable of increasing efficacy without worsening toxicity [15].

Gemcitabine has been shown to be effective and safe as a single agent and in combination regimens and, given its different mechanism of action and partial non cross-resistance with anthracyclines and taxanes, represents an ideal candidate for a three-drug regimen. Such a combination (gemcitabine, epirubicin and paclitaxel – GET) was recently tested in a clinical setting. After very promising results in a phase II study, with a 92% response rate [16], the GET regimen failed to demonstrate a higher efficacy than the FEC (5-fluorouracil, epirubicin and cyclophosphamide) combination in a multicenter randomized phase III trial. In addition to the lack of a significant advantage of GET over FEC in terms of response rate and time to progression, the GET arm showed higher toxicity [17].

We designed a clinical protocol based on a preclinical schedule that had produced the highest synergistic drug interaction in an attempt to improve the clinical efficacy obtained with empirically designed treatment, or at least to achieve similar results but with lower drug dosages and milder toxicity [8,9].

In the present study, we demonstrated that the addition of gemcitabine to doxorubicin and paclitaxel produced clinical results using low doses of the three drugs, with consequently minimal non hematological toxicity. In particular, the lack of cardio- and neurotoxicity is an important advantage because it permits anthracycline and taxane re-treatment. Whilst it is also clear that gemcitabine significantly increased myelosuppression, this toxicity was manageable with primary or secondary G-CSF prophylaxis. It is important to stress that the toxic death in our population occurred before the first administration of gemcitabine, i.e., after one cycle of doxorubicin and paclitaxel given at lower doses than usual.

Activity data appear comparable with those reported in the previously mentioned phase III randomized trials of anthracycline-paclitaxel based chemotherapy in metastatic breast cancer. In these trials [3-9], response rates ranged from 46% to 68% and median time to progression varied from 6 to 9.8 months. The experimentally designed GAT regimen showed a fairly similar efficacy profile in terms of response rate and time to progression, and results were particularly interesting for the small group of stage IIIB patients, who all responded to treatment and the majority of whom were operable.

Difficulties in translating *in vitro *results into clinical practice are inevitable. In our preclinical study [13], the synergistic sequence of doxorubicin → paclitaxel → gemcitabine was defined in two cancer cell lines, BRC-230 and MCF7, characterized by a 100% growth fraction and a doubling time of around 30 hours. The cell kinetics of human breast cancer are considerably different in that the growth fraction is remarkably low and it takes several days for the cell population to double. Moreover, we know that in clinical practice delays may occur in drug administration due to toxicity, patient compliance, or logistic problems, and these timing violations can affect clinical outcome. These and other important issues about translational research remain unsolved and must be addressed in clinical research.

## Conclusion

In conclusion, our results seem to indicate that the GAT regimen is active in anthracycline-naïve metastatic breast cancer and provides a feasible chemotherapeutic option in this clinical setting.

## Abbreviations

MBC, metastatic breast cancer; ANC, absolute neutrophil count; GAT, gemcitabine, doxorubicin and paclitaxel; TTP, time to progression; OS, overall survival; CR, complete response; PT, partial response; SD, stable disease; PD, progressive disease; G-CSF, granulocyte colony-stimulating factor; GET, gemcitabine, epirubicin and paclitaxel; FEC, 5-fluorouracil, epirubicin and cyclophosphamide.

## Competing interests

The author(s) declare that they have no competing interests.

## Authors' contributions

DA and AP conceived the study and participated in its design and coordination. WZ performed the preclinical study, providing the rationale for the trial. ON participated in the design of the study and carried out the statistical analyses. AP, RM, CM, LF and LG were involved in the treatment and follow-up of patients. IM and FZ were responsible for data management. All authors read and approved the final manuscript.

## Pre-publication history

The pre-publication history for this paper can be accessed here:



## Figures and Tables

**Table 1:** Patient and disease characteristics at baseline

**Patient characteristics**	**Patients (n = 33)**	
	**No.**	**%**
**Age**	59 years	
Median (range)	(35–68)	
		
**PS (ECOG)**		
Median (range)	0 (0–1)	
		
**Menopausal status**		
Pre	6	18.2
Post	27	81.8
		
**Stage**		
Locally advanced	7	21.2
Metastatic:		
1 site	9*	27.3
≥ 1 site	17	51.5
		
**Site**		
Breast	12	36.4
Bone	20	60.6
Liver	12*	36.4
Lung	8	24.2
Lymph nodes	4	12.1
		
**Receptor status**		
ER+	22	66.7
ER-	8	24.2
Unknown	3	9.1
		
**Prior therapy**		
None	16	48.5
Adjuvant hormonal therapy	4	12.1
Adjuvant chemotherapy	7	21.2
Chemo- and hormone adjuvant therapy	4	12.1
Hormone therapy for advanced disease	6	18.2

*One instrumentally detected hepatic lesion was histologically diagnosed *a posteriori *as an angioma

**Table 2:** Best tumor response and overall response rate (ORR) by disease site (n = 29)

**Best tumor response**	**No.**	**%**
Complete response	2	6.9
Partial response	14	48.3
Overall response rate	16	55.2 (CI 37.5–62.5)
No change	10	34.5
Progressive disease	3	10.3
		
**ORR by disease site**		
Breast (locally advanced)	9/12	75.0
Viscera	9/13	69.2
Liver	3/11	27.3
Lymph node	6/6	100

ORR, overall response rate

**Table 3:** Treatment compliance

	**n = 169 cycles**	
	**No.**	**%**
Delay 1st day		
1–8 days	3	1.8
9–15 days	3	1.8
> 15 days	1	0.6
		
Delay 6th day		
+1	11	6.5
+2	8	4.7
+3	5	3.0
> +3	4	2.4
		
	**n = 169 cycles**	
	**No.**	**%**

Dose reduction of all drugs		
≤ 25%	18	10.6
26–50%	5	3.0
> 50%	0	0
		
Dose reduction of only A-T		
≤ 25%	3	1.8
26–50%	4	2.4
> 50%	0	0
		
Dose reduction of only GEM		
≤ 25%	2	1.2
26–50%	7	4.1
> 50%	0	0

A, doxorubicin; T, paclitaxel; GEM, gemcitabine

**Table 4:** Toxicity per cycle and per patient

**Toxicity**	**Per cycle (n = 169)**			**Per patient (n = 33)**		
		
**Hematological**	**Grade 2**	**Grade 3**	**Grade 4**	**Grade 2**	**Grade 3**	**Grade 4**
	**No. (%)**	**No. (%)**	**No. (%)**	**No. (%)**	**No. (%)**	**No. (%)**

Leucopenia	51 (30)	31 (18)	9 (5)	11 (33)	6 (18)	8 (24)
Neutropenia	11 (6)	22 (13)	60 (36)	2 (6)	5 (15)	18 (54)
Thrombocytopenia	12 (7)	4 (2)	0	5 (15)	4 (12)	0
Anemia	26 (15)	5 (3)	0	12 (36)	2 (6)	0
						
**Non hematological**						
	**No. (%)**	**No. (%)**	**No. (%)**	**No. (%)**	**No. (%)**	**No. (%)**

Nausea/vomiting	14 (9)	2 (1)	0	9 (27)	1 (3)	0 (0)
Diarrhea	8 (5)	1 (1)	1 (1)	7 (21)	1 (3)	1 (3)
Stomatitis	14 (8)	1 (1)	0	7 (21)	1 (3)	0
Neurosensory	14 (8)	0	0	6 (18)	0	0
Fever	16 (9)	0	0	8 (24)	0	0
Infection	2 (1)	0	0	1 (3)	0	0
Thromboembolism	2 (1)	0	0	2 (6)	0	0
Hypersensitivity	1 (1)	0	0	1 (3)	0	0
